# **CCN5/W**ISP2 and metabolic diseases

**DOI:** 10.1007/s12079-017-0437-z

**Published:** 2017-12-15

**Authors:** John R Grünberg, Johannes Elvin, Alexandra Paul, Shahram Hedjazifar, Ann Hammarstedt, Ulf Smith

**Affiliations:** 1 Metabolic Research Laboratories, Wellcome Trust MRC Institute of Metabolic Science, Addenbrooke’s Hospital, University of Cambridge, Cambridge, CB2 0QQ UK; 2 The Lundberg Laboratory for Diabetes Research, Department of Molecular and Clinical Medicine, the Sahlgrenska Academy, University of Gothenburg, 405 30 Gothenburg, Sweden; 3 Department of Biology and Biological Engineering, Chalmers University of Technology, 41296 Gothenburg, Sweden

**Keywords:** Adipose tissue, Fibrosis, Insulin resistance, Metabolism, Mesenchymal stem cells, WNT-signaling

## Abstract

Obesity and type 2 diabetes increase worldwide at an epidemic rate. It is expected that by the year 2030 around 500 million people will have diabetes; predominantly type 2 diabetes. The CCN family of proteins has become of interest in both metabolic and other common human diseases because of their effects on mesenchymal stem cell (MSCs) proliferation and differentiation as well as being important regulators of fibrosis. We here review current knowledge of the WNT1 inducible signaling pathway protein 2 (CCN5/WISP2). It has been shown to be an important regulator of both these processes through effects on both the canonical WNT and the TGFβ pathways. It is also under normal regulation by the adipogenic commitment factor BMP4, in contrast to conventional canonical WNT ligands, and allows MSCs to undergo normal adipose cell differentiation. CCN5/WISP2 is highly expressed in, and secreted by, MSCs and is an important regulator of MSCs growth. In a transgenic mouse model overexpressing CCN5/WISP2 in the adipose tissue, we have shown that it is secreted and circulating in the blood, the mice develop hypercellular white and brown adipose tissue, have increased lean body mass and enlarged hypercellular hearts. Obese transgenic mice had improved insulin sensitivity. Interestingly, the anti-fibrotic effect of CCN5/WISP2 is protective against heart failure by inhibition of the TGFβ pathway. Understanding how CCN5/WISP2 is regulated and signals is important and may be useful for developing new treatment strategies in obesity and metabolic diseases and it can also be a target in regenerative medicine.

## Multipotential mesenchymal stem cells and their regulation by CCN5/WISP2

Mesenchymal stem cells (MSCs) are self-renewing, multipotent cells with stem cell-like characteristics found in adult tissues. These cells have the capacity to differentiate into multiple cell types with a broad variety of physiological functions and are present in nearly all tissues where they are involved in regeneration and cellular homeostasis.

MSCs from the bone marrow are the most frequently investigated. However, MSCs from other tissues such as the peripheral blood, adipose tissue, cardiac tissue, and perinatal tissues also have potential to proliferate and differentiate into the adipogenic, chondrogenic, and osteogenic lineages and, subsequently, to differentiate into functional cardiomyocytes, endothelial, neural and insulin-producing cells (Hass et al. 2011).

The CCN family of proteins play an important role in MSC regulation and its expression is high in both embryonic and adult tissue. CCNs play an important role during embryonic development, wound healing, injury repair, angiogenesis, and fibrosis and can interact with, and modulate, signals by integrins, BMPs, VEGF, Notch, and canonical WNTs. (Jun and Lau 2011; Klenotic et al. 2016; Zuo et al. 2010). Canonical WNT signaling is of particular importance for the determination of MSC fate and promotes entry of mesenchymal precursor cells into the myocyte and osteocyte lineages while suppressing commitment to the adipocytic lineage and adipose cell terminal differentiation (Armani et al. 2010; Christodoulides et al. 2009; Gustafson et al. 2009; Gustafson and Smith 2010). One of the genes activated by the canonical WNT signaling is the WNT1-inducible signaling pathway protein 2 (WISP2/ CCN5) (Inadera et al. 2009; Longo et al. 2002) (Pennica et al. 1998). *Cnn5/Wisp2* has been shown to be activated by the canonical WNT and not the non-canonical WNT signaling pathways. CCN5/WISP2 has a molecular size of around 27.5 kDa and the homology between mouse and human CCN5/WISP2 is high (73%) (Pennica et al. 1998; Wei et al. 2009). We have also found that human/mouse-CCN5/WISP2 has similar effects both in human and mouse adipose cells in vitro.

While the effects of CCNs are diverse in many tissues, this review will focus on the role of CCN5/WISP2 and its effects in metabolic diseases, in particular obesity and diabetes.

## CCN5/WISP2 and metabolic disease

### Metabolic syndrome

CCN5/WISP2 was previously found by microarray analysis to be one of the genes upregulated in the adipose tissue of First Degree Relatives (FDR) of patients with type 2 diabetes, a very high-risk group for the development of diabetes, Hammarstedt et al. (2013) found the expression of *CCN5/WISP2* to be associated with WNT-regulated genes such as *CYCLIND1, insulin resistance,* and markers of hypertrophic obesity, i.e., increased subcutaneous cell size and waist circumference in non-diabetic individuals. *CCN5/WISP2* was also positively correlated with markers of ectopic fat accumulation (i.e.,fat in liver or non-subcutaneous / intra-abdominal adipose tissue) and negatively correlated with whole-body insulin sensitivity, a marker of risk of developing type 2 diabetes. These data provide evidence for increased activation of canonical WNT in the adipose tissue in the Metabolic Syndrome. *CCN5/WISP2* is highly expressed in mesenchymal stem cells and undifferentiated preadipocytes and CCN5/WISP2 protein is not found in isolated mature adipocytes. During differentiation of both human preadipocytes and murine 3T3-L1 preadipocytes, *CCN5/WISP2* is rapidly downregulated. However, it remains elevated in the adipose tissue in hypertrophic obesity/Metabolic Syndrome as a consequence of the impaired adipogenesis in this condition.

Positive energy balance leads to accumulation of lipids in the subcutaneous adipose tissue but this tissue has a limited expandability and, when exceeded, lipids accumulate ectopically in visceral depots, liver, around the heart, and other organs (Despres et al. 2008; Snel et al. 2012; Virtue and Vidal-Puig 2010). Experimental studies have shown that this can be prevented by a hyperplastic adipogenic response as seen, for instance, in mice overexpressing adiponectin in the adipose tissue. This leads to an extreme obesity, but of a metabolically “healthy” phenotype with many small and insulin- sensitive cells (Kim et al. 2007). Not only obesity, but also lack of adipose tissue as in genetic lipoatrophy, leads to insulin resistance and ectopic fat accumulation, which can be reversed by adipose tissue transplantation to allow the lipids to be stored appropriately (Gavrilova et al. 2000).

*CCN5/WISP2* transcriptional activation is higher in subcutaneous adipose tissue compared to visceral tissue and also higher in the adipose tissue in equally obese individuals fulfilling the criteria for the Metabolic Syndrome. This is likely a consequence of the impaired adipogenesis in this condition rather than inappropriate regulation of *CCN5/WISP2* activation. This is supported by our findings in a genetic mouse model overexpressing *Ccn5/Wisp2* in the adipose tissue with an aP2/FABP4 promoter (aP2-Wisp2) (Grunberg et al. 2017). These mice demonstrated the positive effect of *CCN5/*WISP2 on mesenchymal precursor cell growth and subsequent differentiation. The mice had increased glucose tolerance, insulin sensitivity, and hyperplastic brown and white adipose tissues with more numerous but smaller adipocytes. These results will be further discussed later in this review.

### CCN5/WISP2 and adipogenesis

The ability to recruit and commit MSCs to the adipogenic lineage is crucial for a healthy expansion of the adipose tissue during weight expansion rather than merely enlarging the available cells. Furthermore, there is a 10% annual turnover of the adipose cells in man (Arner et al. 2010). Thus, there is a continuous recruitment of progenitor cells which undergo subsequent differentiation to new adipose cells. CCN5/WISP2 has profound effects on both adipogenic commitment and differentiation of adipocytes (Hammarstedt et al. 2013). Like other CCN proteins (Perbal 2013), CCN5/WISP2 is both present in the cytosol and secreted, and prevents adipogenic commitment and PPARγ-induced differentiation through two different mechanisms. Cytosolic CCN5/WISP2 forms a complex with the PPARγ transcriptional co-activator ZNF423 (Gupta et al. 2010). This prevents ZNF423 from entering the nucleus and activating transcriptional programs that allow the cells to enter commitment to the adipocyte lineage. The CCN5/WISP2-ZNF423-complex is dissociated by BMP4 through the SMAD binding domain on ZNF423 which then allows its nuclear entry. BMP4 is also an important inhibitor of ZNF423 in mesenchymal progenitor cells (Grunberg et al. 2014). Together, these are important mechanisms for adipogenic commitment of mesenchymal progenitor cells as also shown by the induction of adipogenic markers when CCN5/WISP2 is silenced by BMP4. The secretion of BMP4 inhibitors such as Gremlin 1 in human cells (Gustafson et al. 2015) or Noggin in murine cells (Gustafson and Smith 2012) prevents the ability of BMP4 to dissociate the CCN5/WISP2-ZNF423-complex and as a consequence, also adipogenic differentiation. The adipose tissue secretes endogenous BMP4, and this is enhanced in obesity, in order to promote the recruitment of new progenitor cells rather than merely expanding available cells and developing a dysfunctional hypertrophic obesity. The importance of the endogenous and secreted BMP inhibitors in preventing BMP4-induced precursor cell adipogenic commitment and differentiation and developing an adipose tissue BMP4 resistance has been shown in both human (Gustafson et al. 2015) and murine cells (Hoffmann et al. 2017).

Secreted CCN5/WISP2 promotes proliferation of mesenchymal precursor cells but also inhibits their adipogenic commitment and differentiation (Grunberg et al. 2014; Hammarstedt et al. 2013). Like the canonical WNT3a ligand (Gustafson and Smith 2010), it activates the canonical WNT pathway and prevents PPARγ-activation. Thus, CCN5/WISP2 is not only induced by canonical WNT activation but it also, in part, signals through the same pathway. Secreted CCN5/WISP2 initiates transcriptional activation of *Tcf/Lef* and directs β-catenin to the nucleus, whereas silencing of *Ccn5/Wisp2* leads to a decrease in β-catenin as well as its nuclear-targeted phosphorylation. The specific receptor for CCN5/WISP2 is currently unknown but is it unlikely to be a member of the Frizzled family of receptors as discussed below.

### CCN5/WISP2 receptor and signaling

The specific CCN5/WISP2 receptor is currently unknown but LRP5/6 is a potential candidate. LRP5/6 is a co-receptor for canonical WNT and TGFβ as well as several other ligands including CTGF and PDGFα through physical interaction with the cognate receptors (Ren et al. 2013). CCN5/WISP2 does not need acylation for its secretion (Grunberg et al. 2014) while other conventional canonical WNT ligands have to be acylated in order to be secreted and bind to the FZD receptors (Clevers and Nusse 2012; Willert and Nusse 2012). Thus, CCN5/WISP2 may bind to the LRP5/6 receptor directly and/or activate it through other signaling pathways.

Additional supporting evidence that CCN5/WISP2 signals through the LRP5/6 receptor is the finding that the canonical WNT inhibitor DKK1 antagonizes the inhibitory effect of CCN5/WISP2 on *Pparg* and *Fabp4* transcriptional activation (Grunberg et al. 2014). DKK1 is a both a marker and mediator of well-functioning adipogenesis (Christodoulides et al. 2006) and can partly rescue the impaired adipogenesis in hypertrophic obesity further supporting the importance of secreted CCN5/WISP2 in regulating adipogenesis (Gustafson and Smith 2012). How DKK1 and other canonical WNT antagonists are regulated is currently unclear but PPARγ activation can increase the secretion of DKK1 in adipose cells (Gustafson et al. 2010). Once PPARγ is activated it suppresses WNT-activation by increasing the degradation of β-catenin and thus maintaining the differentiated state (Gustafson et al. 2010; Liu et al. 2006).

It has also been shown that CCN5/WISP2 interacts with the cell surface receptor integrin αvβ3 in vascular smooth muscle cells (VSMC) and podosomes, but the downstream signaling effects are unknown. CCN5/WISP2 does, however, prevent the matrix degradation required for cell migration in podosomes (Myers et al. 2014). This is further supported by data from the Castellot laboratory showing that ectopic expression of CCN5/WISP2 in a mouse model for vascular restenosis strongly suppresses VSMC migration and proliferation. It was suggested that CCN5/WISP2 protects against restenosis by blocking the ability of medial VSMC podosomes to degrade matrix, thus preventing migration into the intima (Myers et al. 2014).

Integrin αvβ3 is a promiscuous receptor that binds a wide range of proteins (Myers et al. 2014) and it is possible that CCN5/WISP2 also interacts with integrin αvβ3 to mediate further downstream signaling including MAPK activation. We found both p38 MAPK and ERK MAPKinases to be activated by CCN5/WISP2 in mature adipocytes (Grunberg et al. 2014). However, further studies are needed to clarify the potential cross-talk between CCN5/WISP2 and integrin αvβ3.

### Regulation of CCN5/WISP2

*Ccn5/Wisp2* transcript begins to be expressed at the early medulla stage (12–16 cells) in embryogenesis and it persists in all three germ layers (endoderm, mesoderm, and ectoderm) throughout the embryonic development in mice. The CCN5/WISP2 protein is present in most cells of early embryos and is not restricted to a particular germ layer in mice and humans. Tissue specificity appears as the embryo develops. In adult rodents, CCN5/WISP2 is widely distributed in many cell types, both in the cytosol and the nuclei, but CCN5/WISP2 has not been found in the nucleus of mouse and rat pancreas, liver, or spleen (Gray et al. 2007; Jones et al. 2007; Myers et al. 2012; Wiesman et al. 2010).

Canonical WNT3a and GSK3β inhibition increases *Ccn5/Wisp2* expression (2–3 times) in mesenchymal stem/precursor cells (Hammarstedt et al. 2013), as well as insulin like growth factor 1 (IGF-1) levels in murine pancreatic beta cells (Chowdhury et al. 2014), but the detailed regulation of CCN5/WISP2 is largely unknown. *Ccn5/Wisp2* expression is associated with IGF-1 induced islet cell survival and proliferation. Interestingly, miRNA 450a-5p inhibits both the CCN5/WISP2 mRNA and protein levels in a dose-dependent manner in exosome-like vesicles derived from rat adipose tissue (Zhang et al. 2017).

The CCN5/WISP2 promoter contains *TCF*, hypoxia inducible factor (*HIF)*, and nuclear factor kappa-light-chain-enhancer of activated B cells (*NFκβ*) sequences as well as binding domains for PPARγ and its transcriptional co-activator ZFP423. CCN5/WISP2 is regulated by hypoxia through the HIFα isoforms in low-invasive luminal-like breast cancer cell lines, preferentially by HIF2α. CCN5/WISP2 is also negatively correlated with tumor macrophage invasion in breast cancer samples which could provide an additional marker for a better tumor prognosis (Fuady et al. 2014). CCN5/WISP2 has also been reported to be directly regulated by estrogen in the human breast cancer cell line MCF-7 and non-transformed human mammary epithelial cells, and is more highly expressed in a less-aggressive breast cancer cell line (MCF-7) compared with a highly aggressive (MDA-MB-231) (Banerjee and Banerjee 2012; Inadera 2003; Inadera et al. 2000; Zoubine et al. 2001).

Expression data from 79 human tissues showed that *CCN5/WISP2* is by far most highly expressed in the adipose tissue (upregulated 950 times) (Online_database_BIOGPS 2015). Similar to the findings by Hammarstedt et al. (Hammarstedt et al. 2013), the secretome of human adipose tissue was analyzed and showed that CCN5/WISP2 is a highly secreted adipokine that is downregulated in the visceral adipose tissue, compared with the subcutaneous adipose tissue, and correlated to obesity (Dahlman et al. 2012). Furthermore, *CCN5/WISP2* expression has been implicated to be a marker of number and/or activity of adipose precursor cell populations and extracellular matrix remodeling in cattle and a good predictor of intramuscular fat, i.e., marbling of the meat that impacts flavor and juiciness (Hudson et al. 2014).

The in vivo effects of CCN5/WISP2 in the adipose tissue have been studied using the aP2-Wisp2 mice (Grunberg et al. 2017). The aP2-Wisp2 mice showed a completely different phenotype compared with other in vivo models studying the metabolic consequences of canonical WNT. WNT10b overexpression under the aP2-promoter displayed an obesity-protected phenotype with reduced brown and white adipose tissue, reduced weight and the mice were not insulin resistant (Wright et al. 2007). Overexpressing activated β-catenin in PPARγ-expressing adipose precursor cells showed a similar lipodystrophic phenotype while using the later aP2-promoter in differentiated cells did not produce a clear phenotype. Moreover, mice overexpressing β-catenin in the precursor cells were found to release unidentified factor(s) that increased glucose uptake in muscles ex vivo (Zeve et al. 2012).

Transgenic aP2-Wisp2 mice (Grunberg et al. 2017) (Tg) on high fat diet (HFD) had similar body weight and were more insulin-sensitive during both non- and steady state conditions and this was also validated ex vivo. There were several markers of increased mesenchymal tissue growth such as increased and hyperplastic BAT, lean body mass, and weight of skeletal muscles/heart. Serum from Tg mice promoted proliferation of mesenchymal precursor cells and this effect was inhibited by CCN5/WISP2 monoclonal antibodies, verifying the direct proliferative effect of elevated levels of CCN5/WISP2 in the circulation.

**Fig. 1:**
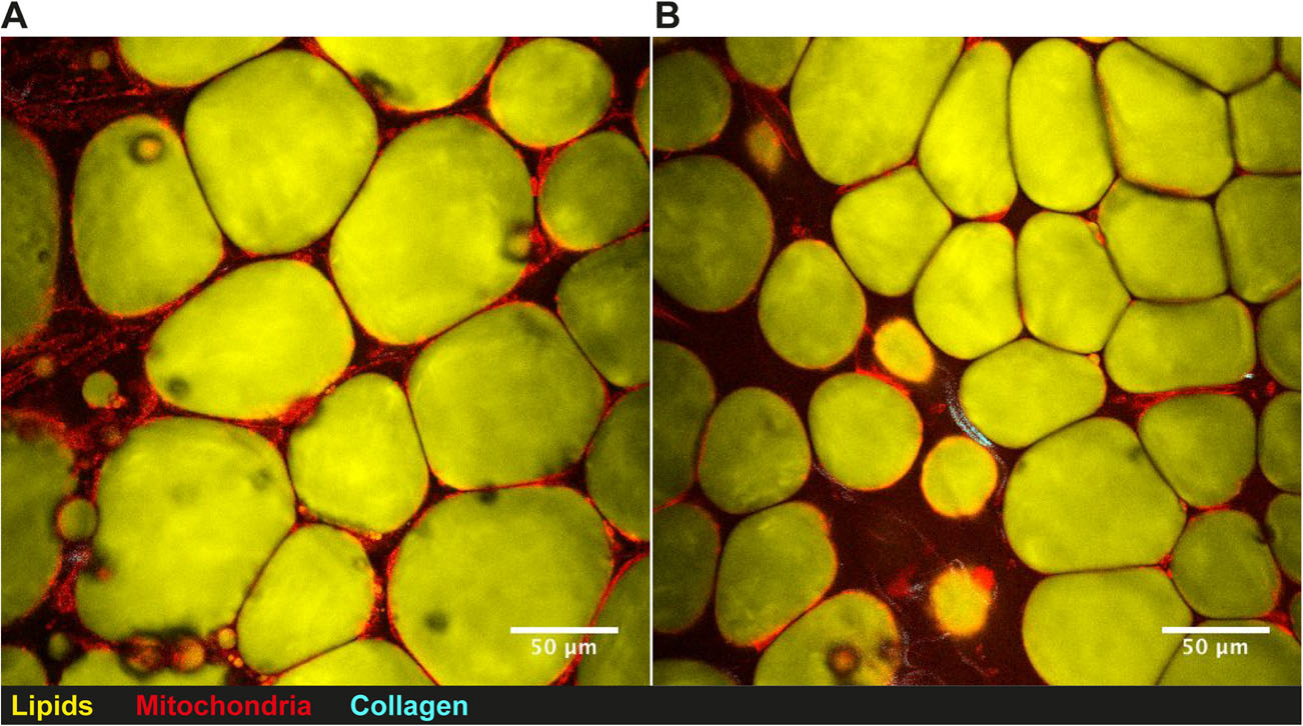
**Subcutaneous white adipose tissue visualized by nonlinear microscopy.** Subcutaneous adipose tissue from (**a**) 19 week old control Black6/N mouse and (**b**) transgenic aP2-Wisp2 littermate.. Mice were fed high-fat diet for 12 weeks prior to termination and CARS analysis of adipose tissue. Mice were terminated and freshly isolated adipose tissue was stained with Rhodamine 123 for active mitochondria and analysed while being kept hydrated at 37 °C. A custom built coherent anti-Stokes Raman scattering (CARS), second harmonic generation (SHG), and two-photon excited fluorescence (TPEF) microscope was used to visualize lipids, collagen, and active mitochondria within the adipose tissue, respectively. Lipids were detected via the 2845 cm^−1^ symmetric CH_2_ stretching vibration. All signals were passed through matching bandpass filters and collected on single photon counting detectors. Lipid droplet analysis from CARS images has been described previously (Brannmark et al. 2014)

**Fig. 2:**
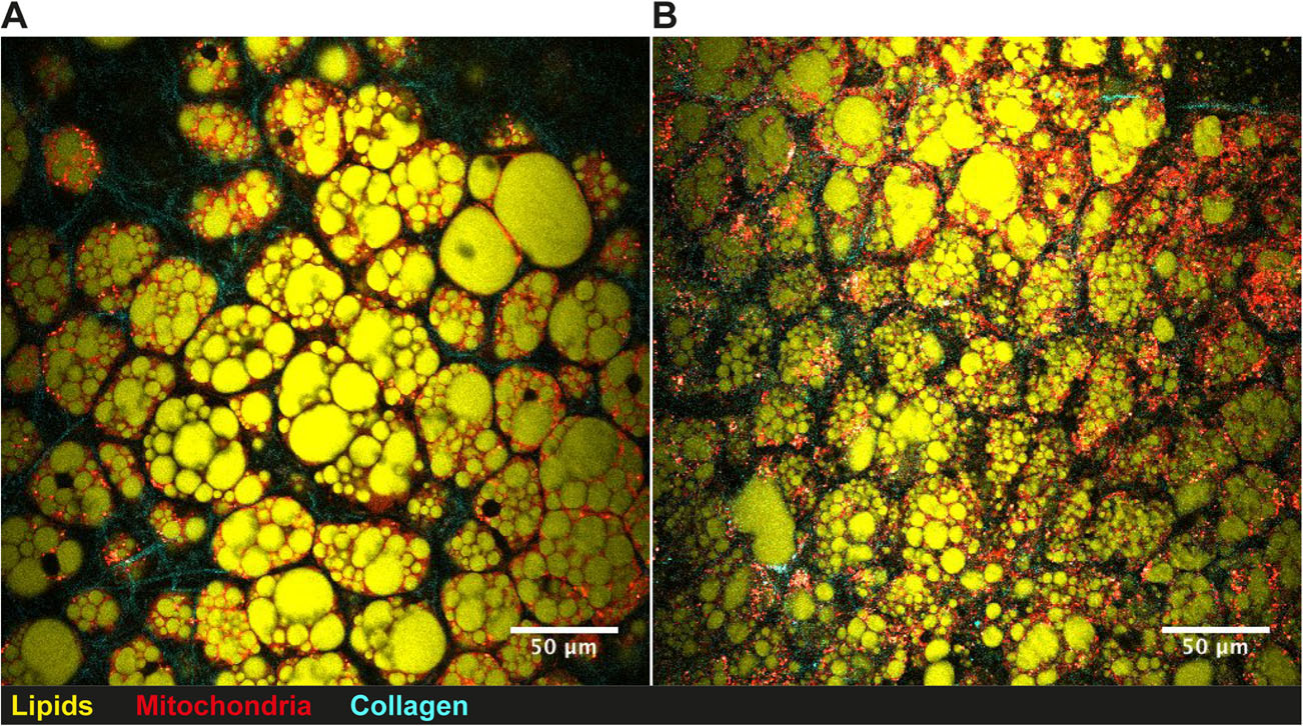
**Brown intrascapular adipose tissue visualized by nonlinear microscopy.** Brown intrascapular adipose tissue from (**a**) 19 week old control Black6/N mouse and (**b**) transgenic aP2-Wisp2 littermate. Mice were fed high-fat diet for 12 weeks prior to termination and CARS analysis of adipose tissue

During HFD in mice, both subcutaneous (sWAT) and epididymal adipose tissue (eWAT) starts to expand through hypertrophy during an early stage. After prolonged caloric excess (1 month), the eWAT initiates adipogenesis, i.e., hyperplasia, which is not seen in the sWAT depots (Wang et al. 2013). However, sWAT in the Tg mice was hyperplastic and characterized by smaller cells, both by mean cell size and total distribution (Fig. 1). This “healthy” adipose tissue profile can probably account for the finding that the Tg mice were more insulin sensitive and had higher circulating adiponectin levels as well as transcriptional activation in the adipose tissue. However, there were no signs of increased beige markers in the white adipose tissues (*Tbx1*, *Tmem26,* or *Cd137*) that could dissipate energy or improve insulin sensitivity (Harms and Seale 2013; Park et al. 2014; Wu et al. 2012). The increased hypercellular BAT mass (Fig. 2) did not show markers of increased activity (unpublished data) with either cold-exposure or a β3-agonist. Thus, the improved insulin sensitivity is most likely associated with positive metabolic effects of the increased lean body mass combined with a “healthy” hyperplastic adipose tissue with increased levels adiponectin and adipose tissue glucose metabolism.

The increased levels of the glucose transporting protein *Glut4* in both adipose tissue and skeletal muscle can be mechanistically related to the increased insulin-stimulated glucose uptake. Increased GLUT4 in the adipose tissue is associated with increased whole-body insulin sensitivity (Herman et al. 2012). Mice overexpressing GLUT4 under the aP2-promoter have recently been shown to also have increased de novo lipogenesis (DNL) regulated by carbohydrate-responsive-element-binding protein (ChREBPβ) (Herman et al. 2012; Ussar and Tschop 2014; Yore et al. 2014). ChREBP is activated by glucose, independent of insulin, and is one of two major transcription factors for DNL. The other is SREBP-1, which is activated by insulin (Lodhi et al. 2011; Xu et al. 2013). Activation of DNL by ChREBPβ in GLUT4 mice leads to increased induction and secretion of lipid species that are metabolically beneficial, called fatty acid esters of hydroxyl fatty acids (FAHFAs), by the adipose tissue (Yore et al. 2014). *Chrebp* was increased in both adipose depots as well as other members important for the DNL (Ussar and Tschop 2014; Yore et al. 2014).

Consequently, we measured several of the novel FAHFAs in serum and found that obesity induced by HFD was associated with lower 13/12- and 5-PAHSA while the levels in the Tg HFD mice were at least as high as in the non-obese mice. This finding can be a likely mechanism for the increased insulin sensitivity in the HFD Tg mice since FAHFAs also increase glucose uptake (Yore et al. 2014). It is unclear how increased CCN5/WISP2 leads to increased levels of FAHFAs but the “healthy” and hypercellular adipose tissue is a likely contributing factor.

To what extent CCN5/WISP2 - FAHFAs can be related to the unknown circulating factor(s) mediating the increased glucose uptake seen in mice overexpressing β-catenin in the precursor cells (Zeve et al. 2012) is currently unclear.

Secreted CCN5/WISP2, under the control of the aP2-promoter in mice (Grunberg et al. 2017), leads to increased amount of BAT and hyperplastic subcutaneous adipose tissue as shown in Fig. 1. This is completely opposite to the results seen in aP2-Wnt10b or the aP2-activated-β-catenin mice models as discussed (Wright et al. 2007; Zeve et al. 2012). This clearly indicates that the proliferative effect of CCN5/WISP2, albeit being a canonical WNT activator in the cell studies (Grunberg et al. 2014), also allows the hyperplastic precursor cells to enter adipogenesis and undergo differentiation. In mesenchymal precursor cells, BMP4 can rapidly inhibit *Ccn5/Wisp2* transcriptional activation but had no acute effect on the conventional canonical WNT activator *Wnt10b* (Grunberg et al. 2017). *Bmp4* expression was increased ≈165% in sWAT and eWAT as well as BAT in Tg mice which may be secondary to the increased adipogenesis which increases cellular BMP4 (Gustafson et al. 2015). However, this finding adds another dimension to the cross-talk between BMP4 and CCN5/WISP2, where BMP4 is a negative regulator of CCN5/WISP2 expression, but not of the canonical WNT10b, and thereby allows the expanded mesenchymal precursor cells to enter normal adipogenic commitment and differentiation. As discussed, BMP4 is secreted by differentiated pre/adipocytes (Gustafson et al. 2015) and acts as a feed-back regulator, promoting the entry of mesenchymal precursor cells into adipogenic commitment and differentiation (Bowers et al. 2006; Gustafson and Smith 2012).

Taken together, CCN5/WISP2 is an endogenous and secreted auto/paracrine non-conventional WNT ligand, targeting mesenchymal precursor cells and promoting their growth and expansion.

CCN5/WISP2 plays several roles in the regulation of adipogenesis by both promoting precursor cell proliferation and tissue growth, by regulating precursor cell commitment in response to BMP4 as well as the subsequent differentiation and PPARγ induction. In addition, as a secreted molecule, CCN5/WISP2 can exert autocrine, paracrine, and also endocrine regulation and be an important adipokine mediating cross-talk between the adipose tissue and other cells. In order to induce adipogenesis, CCN5/WISP2 has to be inhibited by external signals and the key adipose progenitor cell commitment factor BMP4 also inhibits CCN5/WISP2(Grunberg et al. 2017).

Thus, CCN5/WISP2 is a novel regulator of mesenchymal tissue growth and development and can, thereby, also be an important target for preventing obesity- related metabolic complications.

### CCN5/WISP2 is anti-fibrotic in contrast to CTGF

The expanded adipose tissue in hypertrophic obesity is characterized by increased tissue fibrosis. However, fibrosis was not increased in the aP2-Wisp2 Tg mice (Grunberg et al. 2017), possibly because of the hyperplastic adipose tissue with smaller adipocytes in the subcutaneous depots (Fig. 1). A heart muscle-specific CCN5/WISP2 overexpressing mouse model, using α-myosine heavy chain as promoter, further supported the anti-fibrotic effect of CCN5/WISP2. CCN5/WISP2 was shown to protect from cardiac hypertrophy and fibrosis in response to pressure overload when compared to a CTGF-overexpressing model (Yoon et al. 2010). If this is because CCN5/WISP2 does not directly induce fibrosis or if it does not enhance the TGFβ-pathway like CTGF is unknown (Parada et al. 2013; Yoon et al. 2010). The activation of both the canonical WNT and TGFβ signaling pathways have been shown to be required for induction of fibrosis (Akhmetshina et al. 2012).

There is mounting evidence that CCN5/WISP2 is anti-fibrotic and counteracts the effects of several fibrotic markers such as CTGF and α-SMA (Xu et al. 2015a; Xu et al. 2015b). In rat scar tissue, where epidural fibrosis was examined, CTGF was upregulated while CCN5/WISP2 was downregulated on both mRNA and protein level. Overexpression of *Ccn5/Wisp2* in rat fibroblasts from tail skin diminished expression of the myofibroblast marker α-SMA and total collagen concentrations as well as collagen type 1α1 (COL1A1) were decreased. This supports that *Ccn5/Wisp2* inhibits fibroblast to myofibroblast transition (Xu et al. 2015b), which also was shown in human lung fibroblasts (Zhang et al. 2014) and murine cardiac fibroblasts (Jeong et al. 2016).

Furthermore, overexpression of *Ccn5/Wisp2* in human primary skin fibroblasts reduces TGFβ1-induced activation of CTGF as well as their proliferation and differentiation (Xu et al. 2015a).

Both CTGF and CCN5/WISP2 belong to the CCN family of proteins and have a very similar structure. However, unlike the rest of the family members CCN5/WISP2 lacks the cysteine knot (CT) domain. By fusing CCN5/WISP2 with the CT-domain, CCN5/WISP2 gained CTGF-like properties in the same fibroblasts. These results further demonstrate the opposing effects of CCN5/WISP2 and CTGF (Xu et al. 2015a).

The anti-fibrotic effects of CCN5/WISP2 were further validated in patients with heart failure. TGFβ mediates cardiac fibrosis and *CCN5/WISP2* expression is strongly downregulated in patients with heart failure whereas *CTGF* is increased (Jeong et al. 2016). This was also observed in mice with heart failure from transverse aortic constriction. However, heart specific overexpression of *Ccn5/Wisp2*, through AAV9-viruses, preserved echocardiographic parameters, with inhibition of the TGFβ-pathway and several fibrotic genes (Jeong et al. 2016). Notably, overexpression of *Ccn5/Wisp2* not only prevented, but actually reversed, cardiac fibrosis. Taken together, these data add more evidence to the inhibitory effect of CCN5/WISP2 on TGFβ signaling (Jeong et al. 2016).

The Smad proteins, PI3K/Akt and JNK pathways have been suggested to be involved in TGFβ1 induced fibrosis (Conte et al. 2011). Overexpression of *Ccn5/Wisp2* reduced both Smad2 and JNK phosphorylation induced by TGFβ1 (Zhang et al. 2014). In addition, phosphorylation of Akt1 was reduced and the effects of *Ccn5/Wisp2* overexpression were similar to those of a PI3K inhibitor (LY294002). This indicates that the Smad-independent PI3K/Akt pathway is affected in the inhibitory effects of CCN5/WISP2 on fibrosis and CCN5/WISP2 might exert signaling through Smad6 phosphorylation. TGFβ1 is a key mediator in fibrosis progression by activation of its downstream Smad signaling pathway. However, Smad6 can prevent the phosphorylation of other Smad members and therefore act as a negative regulator of the TGFβ mediated pathway (Imamura et al. 1997) and previous studies have shown that silencing *CCN5/WISP2* expression in the breast cancer cell line MCF-7 decreases Smad6 expression levels (Sabbah et al. 2011). Blocking Smad6 phosphorylation ameliorated the inhibitory effect of CCN5/WISP2 on CTGF. When the Smad6 pathway was blocked using siRNA, cell proliferation was once again increased in *Ccn5/Wisp2*-overexpressing cells following TGFβ1 stimulation (Xu et al. 2015b). CCN5/WISP2 also normalized the increased Akt phosphorylation induced by TGFβ in cardiac fibroblasts, consistent with the findings seen in fibroblasts of the lung (Jeong et al. 2016; Zhang et al. 2014).

## Can CCN5/WISP2 be useful in regenerative medicine?

Because of their multilineage potential, ease of isolation compared with embryonic stem cells, fewer ethical issues, and safer profile in terms of oncogenicity (Ren et al. 2012), MSCs have become of interest in the field of regenerative medicine. An exciting new area of translational research is currently investigating the therapeutic potential of MSCs in tissue repair. MSCs can easily be amplified in vitro while retaining their multipotent potential and are proven safe for autologous transplantation. Furthermore, MSCs are capable of homing to lesion areas and migrate into the injured site guided by chemokines released, which potentially simplify the route of administration (Salem and Thiemermann 2010). Since CCN5/WISP2 enhances growth of mesenchymal precursor cells and induces hyperplastic expansion of mesenchymal tissues in transgenic animals (Grunberg et al. 2017), it may also become a target in regenerative medicine and tissue repair.

It is established that the beneficial outcomes of MSCs transplantation occur through paracrine release of biological factors that affect vascular development, are anti-fibrotic and anti-inflammatory facilitating the endogenous repair process rather than direct engraftment into the recipient tissue.

In fact, studies investigating the effect of MSC transplantation, or other stem cell-like cells, in patients with heart failure have shown that the retention and engraftment of transplanted MSCs in the myocardium is disproportional in size and duration to the functional benefits reported. These indirect effects have been attributed to both cell-cell contact and the production and release of positive endocrine factors (Chen et al. 2017; Gnecchi et al. 2008; Leiker et al. 2008).

Although inflammation is a natural and necessary response by the body to many challenges, excessive or prolonged inflammatory stress is harmful for many tissues, not least in the case of adipose tissue contribution to the Metabolic Syndrome and T2D. Interestingly, studies have shown that MCSs can modulate key inflammatory cells in the innate and adaptive immune system making them less inflammatory and instead induce protective cytokines (Gao et al. 2016; Pers et al. 2015; Wang et al. 2012). Consequently, many of the current MCS-based transplantation studies have been performed with the intention to treat immune disorders and with demonstrated clinical potential (Ren et al. 2012). Substantial progress has also been made using MSC in some neurodegenerative diseases where immunomodulation has played a central role in ameliorating disease symptoms (Volkman and Offen 2017).

The possibilities of MSCs have also generated clinical interest in the field of T2D. The studies involve diabetes-related vascular problems and wound healing, but also autologous transplantation of MSCs to improve insulin secretion in patients with newly diagnosed type 1 diabetes or established T2D. The results have been cautiously positive (Moreira et al. 2017).

Clearly, MSC transplantation and identification of factors that promote endogenous MSC activation and tissue regeneration represent clinically relevant solutions for the treatment of many disease conditions. However, although considerable advances have been made in this area, many issues still need to be clarified before it can be routinely used as a therapeutic option.

In sum, CCN5/WISP2 is a growth factor of MSCs and may become useful for restoring tissue growth after damage and/or as an anti-inflammatory and anti-fibrotic factor in human disease.

## Abbreviations

FDR: First Degree Relatives
MS: Metabolic Syndrome
MSCs: Mesenchymal stem cells
WISP2: WNT1 inducible signaling pathway protein 2
